# Clinical Profile and Course of Patients with Acute Respiratory Distress Syndrome due to COVID-19 in a Middle-Income Region in Mexico

**DOI:** 10.2478/jccm-2024-0022

**Published:** 2024-07-31

**Authors:** José Antonio Villalobos-Silva, Gladis Lucia Acros-López, Gracia Lizbeth Velázquez-Estrada, Camilo José Muñoz-Chavez, German Antonio Aguirre-Gómez, Obed Isaí Aguilera-Olvera, Alfredo Sánchez-González

**Affiliations:** Hospital Regional de Alta Especialidad de Ciudad Victoria, Ciudad Victoria, Tamaulipas, Mexico

**Keywords:** ARDS, COVID-19, critical care, intensive care, pneumonia

## Abstract

**Introduction:**

COVID-19 leads to severe clinical complications that culminate in respiratory failure and acute respiratory distress syndrome (ARDS). Despite reports of some comorbidities that contribute to the development of COVID-19-mediated ARDS, there is great variation in mortality rates among critical COVID-19 patients in the world. To date, no studies have described the incidence and comorbidities associated with ARDS due to COVID-19 in the northeastern region of Mexico.

**Aim of the study:**

To describe patients diagnosed with ARDS due to COVID-19 in the northeastern region of Mexico, as well as its variations in comparison with other regions of the country.

**Material and Methods:**

We conducted a prospective and observational study in a tertiary care center in Northeastern Mexico. All patients diagnosed with SARS-CoV-2 infection and requiring management in the intensive care unit were included. We developed a database that was updated daily with the patients' demographic information, pre-existing diseases, disease severity, and clinical variables.

**Results:**

The incidence of ARDS secondary to COVID-19 in HRAEV was high in comparison with other reports. Diabetes mellitus was the risk factor most associated with COVID-19-mediated ARDS.

**Conclusion:**

Based on our findings and those previously reported in the literature, there are differences that we discuss between northeastern and central Mexico, and analyze other areas evaluated around the world.

## Introduction

Acute Respiratory Distress Syndrome (ARDS) is a heterogeneous clinical entity characterized by inflammatory and necrotizing phenomena in pulmonary alveoli, that spread throughout the body via the circulatory system; most cases warrant assisted mechanical ventilation [1,2]. This syndrome is heterogeneous, and several phenotypes have been proposed according to the predisposing clinical risk factors or whether the lung injury causes were direct or indirect [3,4,5,6,7,8,9]. The incidence and mortality rate of ARDS were low before the COVID-19 pandemic (10% and 35%, respectively) [3,10,11]. ARDS caused by the SARS-CoV-2 virus (COVID-19-ARDS) became the most frequent cause of respiratory failure (≈ 46% of cases due to COVID-19), with an associated variable mortality rate, between 23% and 90% [12,13,14,15,16,17,18,19,20,21,22]. Therefore, considering its mortality rate and the specific treatment requirements, it was necessary to describe its epidemiology and associated risk factors given our country's sociocultural differences and our hospital quality context.

The development of ARDS can be fostered by a predisposing risk factor such as pneumonia, non-pulmonary sepsis, gastric aspiration, trauma, pancreatitis, burns, inhalation injury, drug overdose, multiple transfusions, or shock [2]; in the specific case of ARDS due to COVID, hypertension, obesity, and diabetes mellitus are the preeminent risk factors [23,24,25]. Likewise, a relationship between mortality in patients with ARDS due to COVID-19 and hospitalization time has also been described [23].

Currently, the incidence of individuals infected with SARS-CoV-2 has decreased significantly [26]. However, epidemiologically, the probability that new viruses resulting from SARS-CoV-2 mutations and that now coexist with other respiratory infectious agents or other diseases, is possible: these may potentially unleash complex healthcare scenarios [27]. In addition, most studies on COVID-19 do not describe the injuries resulting from ARDS due to COVID-19 and its risk factors, despite their clinical impact. Most of the information reported has focused on certain parts of the world, and regions such as Latin America have been scantily evaluated; most importantly, the socioeconomic, environmental, and hospital care context in this area differs greatly from the high- and middle-income world [28,29]. Mexico is a clear example since its particular environmental and socioeconomic milieu conditions various scenarios, that impinge on our hospital resources, both human and infrastructural [21,30,31].

Mexico is one of the Latin American countries that has most addressed the issue of COVID-19, but the analyses remain insufficient (1.2% of overall global participation) [21,28]. The first registered case of COVID-19 in Mexico was detected on February 28, 2020, and during the pandemic's peak, Mexico became the fifth country with the most reported cases of SARS-CoV-2 viral infections in America [31]. Nowadays, two studies have reported the incidence of ARDS due to COVID-19 in the central and western regions of the country, as 24% and 40%, respectively [21,22].

ARDS is an entity that can develop for different reasons, so its incidence and mortality must be analyzed in the context of its etiology, particularly resulting from pathologies with a significant incidence such as COVID-19. Specifically, in Northeastern Mexico, the High Specialty Regional Hospital of Ciudad Victoria (HRAEV) was one of the medical centers with the greatest number of COVID-19 admissions since it was designated to become an all-COVID hospital between 2020 and 2022. Thus, it captured a significant number of infected patients in this region. This is a geographical area with a predominantly middle-income population, with cultural, sociodemographic, and hospital infrastructure features that influence the comorbidities and risk factors associated with ARDS due to COVID-19. This study aims to report the incidence of ARDS secondary to COVID-19 in the northeastern region of Mexico in comparison with the findings reported in other regions of the country, as well as the associated sociodemographic and clinical variables.

## Materials and methods

We conducted a prospective and observational study in a tertiary care center in Northeastern Mexico, the *Hospital Regional de Alta Especialidad de Ciudad Victoria* (HRAEV). As a result of the SARS-CoV-2 pandemic, the HRAEV became an all-COVID hospital as of April 2020, and the ICU only managed these patients.

We included all patients above the age of 18 in need of invasive mechanical ventilation. All had an established diagnosis of SARS-CoV-2 bilateral pneumonia confirmed with a swab test and RT-PCR; the pulmonary infiltrates observed in the computerized tomography scan (CT) were analyzed to describe their level of severity. Nevertheless, patients who could not be fully evaluated in terms of diagnosis, treatment, and ARDS prognosis due to treatment withdrawal or early death upon ICU admission, were excluded from the study.

We developed an electronic database that was updated daily with the patients' demographic information, such as age, sex, body mass index, pre-existing diseases, days hospitalized, complications, death, disease severity variables, APACHE - SOFA score, and clinical variables: MAP, HR, RR, ScO_2_, temperature, SvcO_2_, ventilation mechanics parameters, tidal volume, plateau pressure, volume/minute, FiO_2_, total PEEP, PaO_2_/FiO_2_, compliance, alveolar distending pressure, PaO_2_, PaCO_2_, biochemical variables, ferritin, DD, platelets, and inflammation biomarkers. Lung imaging studies were obtained in a SIEMENS 64-cut multidetector CT scanner. It is important to note that, to meet the objective of this study, data reported in the few studies of a similar nature and conducted in other regions of Mexico were also obtained.

### Study definition

Acute respiratory distress syndrome (ARDS) was defined as acutely developing hypoxemia (ratio of arterial oxygen partial pressure and inspired oxygen fraction (PaO_2_ / FiO_2_) <300) with bilateral lung opacities in chest images, and not explained by congestive heart failure. Patients with ARDS required invasive mechanical ventilation and were managed per the airway protection guidelines (tidal volume ≤6 ml/kg, plateau pressure <30 cmH_2_O, and conduction pressure <15 cmH_2_O), and rescue therapy when warranted (prone decubitus, recruitment maneuvers, nitric oxide (NO), and/or extracorporeal membrane oxygenation (ECMO)) [2].

### Statistical analysis

Statistical analysis was based on descriptive measures, percentages, central tendency measures, dispersion measures for quantitative variables, and frequency distribution measures for qualitative variables.

We used the SPSS 24 statistical package [32], the Chi-square test for categorical variables, and Student's t-test for continuous variables. A *p* value < 0.05 was considered statistically significant [33].

### Case presentation/presentation of case series

In this study, patients diagnosed with ARDS due to COVID-19 were admitted to the intensive care unit (ICU) when requiring mechanical ventilation. To ensure the correct diagnosis and severity of ARDS, the universal Berlin criteria were applied before admission. Subsequently, the patient’s sociodemographic data, respiratory parameters, and laboratory variables were recorded, from the time the patient was admitted until discharge or death.

### Ethics

The study was approved by the Ethics and Research Committees of the *Hospital Regional de Alta Especialidad de Ciudad Victoria* (Registration folio: HRAEV-IC-002-23). Informed consent was obtained from the family for the subsequent use of the patient's information, and full anonymity was assured to all participants.

## Results

**Fig. 1.**
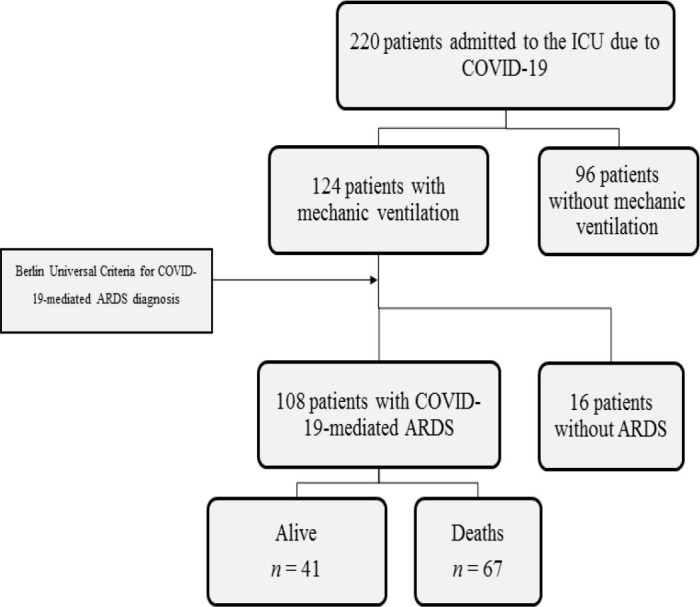
220 COVID-19 patients were treated in the hospital during the study period

**Fig. 2.**
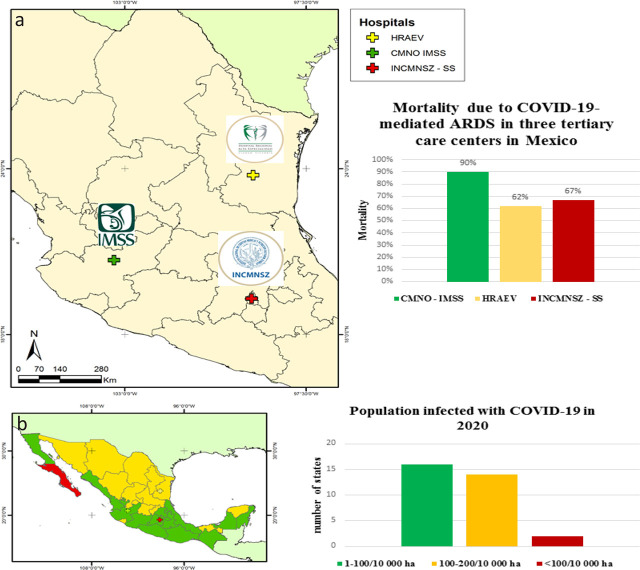
a) Map of the mortality (percentage) due to COVID-19-mediated ARDS in three tertiary care hospitals in Mexico; b) Map of the number of infected individuals per 10,000 inhabitants in the Mexican Republic.

Between early April 2020 and January 2021, 220 critical patients were admitted to the intensive care unit with a PCR-confirmed COVID-19 diagnosis and bilateral pneumonia requiring high-flow oxygen therapy. In this cohort, 124 patients had severe respiratory failure warranting mechanical ventilation in the first 24 hours after admission, and therefore prone to develop COVID-19-related ARDS. Per the Berlin universal criteria, 108 patients (49%) were diagnosed with ARDS due to COVID-19 and represented the cohort upon which this study is based (Figure 1). This significantly differs from the findings reported in two other tertiary care hospitals in Mexico, located in different regions and with a different epidemiological context (Table 1 and Figure 2). When compared with our cohort, mortality from COVID-19-mediated ARDS also revealed significant differences. (Table 1 and Figure 2).

**Table 1.** Incidence and mortality parameters of patients with ARDS due to COVID-19 in three tertiary care hospitals in the Mexican Republic. (N = number of patients infected by COVID-19) *[22] **[21]

**Hospital**	**N**	**ARDS (%)**	**Chi-2 Test**	**Mortality (%)**	**Chi-2 Test**	**Average age**	**Beds**	**COVID-19 Specialists**	**Comorbidity**
HRAEV	220	108 (49%)	X^2^ =3255.13	67 (62%)	X^2^ =45.04	59	50	13	Diabetes
*CMNO – IMSS	1010	408 (40%)	df=2	364 (90%)	df=2	58	249	≈50	Hypertension
**INCMNSZ	800	241 (30%)	*p* < 0.05	159 (66%)	*p* < 0.05	52	168	≈36	Obesity

### Comparison of live and deceased patients due to ARDS

#### Demographics and Score values

The percentage of live and deceased patients was 35% (n=41) and 62% (n=67), respectively. Mortality was higher in males, and the incidence of death was greatest in individuals between the ages of 55 and 77 (Table 2). However, seventy-five percent (75%) of patients had at least one comorbidity, and the most frequent was systemic arterial hypertension (57%). However, type 2 diabetes mellitus was the comorbidity that conditioned a significant statistical difference between live and deceased patients due to COVID-19-ARDS (Table 1). All cases were evaluated with the “Acute Physiological and Chronic Health Evaluation II” (APACHE II) score, which yielded a mean value of 23±7.5, and the Sequential Organ Failure Assessment (SOFA) score, with a mean of 9±3 (Table 2).

**Table 2.** Patient demographics and comorbidities at baseline

**Variable**	**All (n= 108)**	**Live Patients (N=)**	**Deceased Patients (N=)**	**p-value**
Age (*N*)	59±13	52±12.6	66±11.5	0.00**
Male	72 (67%)	27 (25%)	45 (42%)	*0.88 (NS)*
Female	36 (33%)	14 (13%)	22 (20%)	
Body mass index, kg/m^2^ (*n*)	32.8±6.2	32±6.8	32±5.9	*0.79 (NS)*
Systemic Arterial Hypertension	62 (57%)	23 (21%)	39 (36%)	*0.05 (NS)*
Diabetes Mellitus 2	56 (52%)	19 (17%)	37 (34%)	0.04*
Heart Disease	12 (11%)	5 (4%)	7 (6%)	*0.78 (NS)*
Chronic Renal Failure	6 (5.6%)	2 (1%)	4 (3%)	*0.67 (NS)*
Number of patients with COPD (Chronic obstructive pulmonary disease)	52/108	17/42	35/66	

**Scores**				
NUTRIC-2	6.5±2.5	6.0±1.5	7±2	*0.17 (NS)*
SOFA	9±3	6.8±3.1	10.4±2.3	*0.06 (NS)*
APACHE II	23±7	18±7.6	26±5.3	0.017*

#### Respiratory and diagnostic parameters

The average number of days on mechanical ventilation was significantly different between live and deceased patients. The live patient group required fewer days on assisted ventilation in comparison with the deceased group (19±5 and 27±11, respectively); there was also a difference in the average number of days with COVID-19 before hospital admission between the live and deceased patients (11.1±5.2 and 17±6.7 days, respectively) (Table 3).

**Table 3.** Respiratory variables in patients who developed ARDS due to COVID-19

**Ventilatory parameters**	**All (n=)**	**Live patients (N=)**	**Deceased patients (N=)**	**p value**
Days from symptom onset to mechanical ventilation	14±4.8	11±5.2	17±6.7	0.005**
Days on mechanical ventilation since IUC admission	23±8	19±5	27±11	0.004**
PaCO_2_ (mmHg)	55±14	48±11(45–87)	65±14 (55–105)	0.048*
FiO_2_ %	62±13 (50–90)	55±12 (35–70)	70±15 (50–100)	0.061
Plateau pressure (cm/H_2_O)	28.2±6.2	27.3±5(15–38)	29.5±7.3(16–49)	*0.072*
PaO_2_/FiO_2_ (mmHg)	115±66	153±57(66–304)	126±77(43–400)	0.045*
PEEP (cm/H_2_O)	13.6±2	13±1.7(8–14)	13±3.2(8–18)	*0.781*
Driving pressure cm/H_2_O	15±4 (11–38)	13±5(11–22)	18±7(11–38)	0.05*
Distensibility (ml-cm/H_2_O)	49±17 (13–76)	48±13 (36–76)	25±18(13–43)	*0.061*
Vt-predicted weight	7.2±1.7	6.8±1.2(5.5–7.1)	6.5±0.8(5.6–6.8)	*0.23*
TAC (pattern %) *. targeted	*70%	85%	*50%	*0.24*
Ç. CONsolidated	Ç50%	30%	Ç75%	0.067

The deceased patient group showed significant increases in PaCO_2_, PaO_2_/FiO_2_, and distending pressure (transpulmonary pressure) in comparison with the average normal values. CT scans revealed no significant imaging differences between live and deceased patients (Table 3). Finally, significant laboratory abnormalities were increased ferritin levels, D-Dimer, and the number of peripheral lymphocytes in deceased patients (Table 4).

**Table 4.** Patient diagnostic data, vital signs, and laboratory values at baseline

**Vital signs**	**All (n=)**	**Live Patients (N=)**	**Deceased Patients (N=)**	**p value**
Mean blood pressure, mmHg	80±7	82±9 (65–88)	75±8(59–78)	*0.12*
Heart rate, bpm		97±12 (78–123)	97±11 (78–125)	*0.83*

**Laboratory findings**				
Ferritin (ng/mL)	576±59	597±27 (345–789)	965±32 (489–1450)	0.01**
d-Dimer (ng/mL)	3739±1214	2905±975	6642±1868	0.01**
CRP (mg/dL)	107±23	89±35 (34–123)	116±45 (78–230)	*0.07*
Lymphocytes (%)	08±04 (01–16)	12±04 (05–19)	06±03 (01–20)	0.05*
Leukocytes (10^9^/L)	19.4±23.7	17.6±18.5 (12–28)	24.5±23.7 (13–34)	*0.10*

## Discussion

In this study, the incidence of ARDS secondary to COVID-19 was high when compared to that reported by two other tertiary care hospitals in Mexico, both located in different regions and a different epidemiological milieu (Table 1 and Figure 2). These differences may be caused by the age and comorbidities of the patients infected with COVID-19, a finding that has been widely reported elsewhere [21,22]. Of these two factors, age is notably the factor that could be most related to the development of ARDS due to COVID-19; its incidence percentage, from highest to lowest, is consonant with the average patient age (Table 1). However, whether a specific comorbidity could have a greater influence on the development of COVID-19-ARDS remains to be determined. All three comorbidities may promote the development of COVID-19-ARDS [34,35].

The COVID-19-ARDS mortality in this study was low in comparison with the other two tertiary care hospitals in Mexico (Table 1 and Figure 2). This suggests that comorbidities are not significantly linked to the development of ARDS secondary to COVID-19 but do affect mortality; systemic arterial hypertension is the most significantly correlated pathological entity (Table 1).

Younger patient survival was superior to that in our older group of patients, and there was no difference in body mass index between both groups (Table 2). Age and body mass index are important factors that act upon respiratory system mechanics, leading to structural changes of the chest wall and of pulmonary physiological features such as elastance and compliance, abnormal ventilation and gas exchange, decreased exercise tolerance, and decreased respiratory muscle strength [36]. These values are consistent with previous publications by the *CMNO: IMSS* and *INCMNSZ* hospitals. Age was not a significant factor in the *INCM* study since their survivors and deceased patients were of similar age, ranging between 49 and 51 years. The *CMNO-IMSS* and *HRAEV* hospitals reported significant differences in the mean age of the deceased group (>60 years). Although the body mass index was similar in both groups presented in our study, it is a significant variable since it may compromise respiratory mechanics and mortality; this absent difference may be due to the low number of included patients, and the fact that our population, in general, has an overall increased body mass index, and less patients with a normal body mass index were included.

Diabetes mellitus was the most significant comorbidity differentiating COVID-19-ARDS live and deceased patients. Patients with diabetes are inclined to become infected because the effectiveness of their immune system is hindered by impaired phagocytic cell capabilities [37]; in addition, an elevated level of the ACE-2 receptor has been causally related to diabetes [38], and considering the high mutation rate of the virus and the appearance of new and aggressive strains of SARS-CoV-2 virus, diabetic patients remain at risk of contracting the infection due to the viral affinity to the ACE-2 receptor [39]. These infections also have an increased probability of conditioning dire scenarios in the more susceptible diabetic population.

The average period, measured in days, from symptom onset to mechanical ventilation initiation in patients who developed ARDS due to COVID-19 was 14 days, albeit with a significant difference between live and deceased patients (Table 2). The probability of death decreased in mechanically ventilated patients over a shorter than average period; the average number of days on assisted ventilation in the ICU also played a significant role (Table 2), as reported in publications from other countries [23,40,41,42]. Timely intubation and the prompt initiation of mechanical ventilation once the work of breathing was excessive, and placement of the patient in the prone position appear to decrease mortality [23,43,44]. This is a congruent observation since the ARDS outcome hinges on the severity of lung injury in the first 24 hours after ARDS onset [29]. Primary care is recognized as an essential platform to address the growing burden of chronic disease and to detect and manage infectious disease outbreaks in the most vulnerable areas, but always in a timely manner [27].

Some respiratory variables that reflect the efficacy of alveolar ventilation such as the partial pressure of carbon dioxide (PaCO_2_), the ratio between the arterial blood oxygen partial pressure and the inspired oxygen fraction (PaO_2_/FiO_2_), and the distending pressure were significantly different between live and deceased patients (Table 2); when abnormal, these variables have been associated with increased mortality in ARDS patients [13,23,45,46,47]. These parameters explain the scenario mediating patient death in ARDS due to COVID-19: hypoventilation due to elevated CO_2_ in arterial blood normally increases pulmonary contractility but it becomes constrained in ARDS due to its characteristic lung injury (stiff lung). The pathology features reported in individuals deceased due to severe ARDS secondary to coronavirus infection support these observations; *post-mortem* biopsies reveal bilateral diffuse alveolar injury with cellular fibromyxoid exudates, pulmonary edema with pneumocyte sloughing, and the formation of hyaline membranes [48].

The laboratory variables analyzed in this study showed a significant difference in d-Dimer levels between live and deceased cases, whereby the value was greater in the deceased group (Table 3). Recent publications have reported that elevated d-Dimer concentrations are significantly associated with mortality in patients with ARDS resulting from COVID-19 when also associated with decreased pulmonary function values in the static respiratory system [49].

## Conclusion

HRAEV is one of the most important medical centers in the northeastern region of Mexico and during the pandemic, it captured a significant number of COVID-19 patients; this allowed a representative evaluation of the COVID-19 pandemic scenario in the northeastern region of the country, as well as the consequences and course of COVID-19-mediated ARDS. In this context, we established that despite the high incidence of ARDS due to COVID-19, the mortality rate was not high.

However, our study also confirmed that ARDS caused by COVID-19 is a more dangerous entity compared with ARDS secondary to other disease entities. It is therefore essential to apply new preventive and hospital management strategies that prevent the spread of COVID-19 and the development of ARDS; early respiratory assistance managed by trained professional personnel is pivotal and may contribute to a favorable patient outcome.

Finally, we must underscore the fact that despite the complicated scenario that the HRAEV faced during the pandemic, it is a tertiary care medical facility caught at the crossroads of other socially adverse conditions i.e. its proximity to the US border – a country with a high incidence of COVID-19, its consequent constant migratory activity, and its middle-income economic framework, all entailing certain limitations in infrastructure and adequately trained personnel. However, we were able to provide favorable hospital care and maintain a relatively low mortality rate in comparison with most of the country.
